# An explainable machine learning model predicts pediatric varicella encephalitis

**DOI:** 10.3389/fcimb.2026.1759109

**Published:** 2026-04-15

**Authors:** Xiaoxiao Liu, Danlei Mou, Chongyang Yin, Bo Liu, Kaihua Dong, Chunjie Guo, Yang Zhao

**Affiliations:** 1 Pediatric Department of Beijing You ‘an Hospital affiliated to Capital Medical University, Beijing, China; 2 Department of Infectious Diseases, Beijing You ‘an Hospital, Capital Medical University, Beijing, China; 3 Department of Infectious Diseases, Beijing Tongren Hospital Mentougou Campus, Capital Medical University, Beijing, China; 4 Emergency Department of Beijing You ‘an Hospital affiliated to Capital Medical University, Beijing, China; 5 Pediatric Department of Beijing Di ‘tan Hospital affiliated to Capital Medical University, Beijing, China

**Keywords:** machine learning, model, pediatric, varicella encephalitis, varicella-zoster virus

## Abstract

**Background:**

Pediatric varicella encephalitis is a rare but serious complication of varicella, which has a significant impact on patient prognosis. Early clinical diagnosis is still challenging due to atypical clinical symptoms and lack of specific biomarkers. This study aims to establish a predictive model for pediatric varicella encephalitis and provide a practical tool for early clinical identification of such patients.

**Methods:**

A retrospective analysis method was used in this study. A total of 201 children with varicella were enrolled, including 156 in the training group and 45 in the testing group. LASSO regression, XGBoost and random forest algorithm were used to screen key features, and prediction models were constructed based on 6 algorithms. The discrimination, calibration and clinical applicability of the models were verified by the testing set. Shapley additive interpretation (SHAP) analysis was used to interpret the models.

**Results:**

Six characteristic variables associated with pediatric varicella encephalitis were screened out, among which the random forest model showed excellent predictive performance with an area under the curve of 0.950 (95% confidence interval: 0.948-0.952). The calibration curve confirmed that the model was well calibrated, and decision curve analysis showed that it had high clinical utility and provided the greatest net benefit within the risk threshold range. SHAP analysis showed that rash duration, headache, and vomiting were the main characteristics affecting the occurrence of varicella encephalitis in children. In addition, the study created a clinical web application for real-time risk stratification of patients and personalized risk contributions visualized through SHAP.

**Conclusion:**

This study identified 6 important clinical variables of pediatric varicella encephalitis, and the constructed random forest model can accurately and rapidly identify children with varicella encephalitis, which has important clinical application value for early clinical intervention.

## 1 Introduction

Varicella, caused by the varicella-zoster virus, represents a highly contagious disease belonging to the human alpha-herpesvirus family (Zerboni et al., 2014; Kennedy and Gershon, 2018; Han et al., 2025; Paião et al., 2025). Epidemiological data indicate an average annual incidence rate of 44.8 cases per 100,000 population in China, with children aged 5–14 years constituting the primary affected group, accounting for over 50% of all cases (Xuan et al., 2024). Characteristic of the disease is a pruritic, vesicular rash that predominantly distributes over the trunk, head, and face. Accompanying symptoms may include malaise, fever, and fatigue, with the entire course typically lasting about one week. Although the prognosis is generally favorable in most cases, varicella can lead to various severe complications, such as bacterial skin infections, encephalitis, and pneumonia (Kennedy and Gershon, 2018, Poussier et al., 2024). Among these, varicella encephalitis, though relatively uncommon, may result in permanent brain damage, disability, or even fatal outcomes if not promptly diagnosed and managed (Lizzi et al., 2019; Andrei and Snoeck, 2021; Le et al., 2023; Hakami et al., 2024). Therefore, early identification and intervention are of great clinical significance for preventing the adverse consequences of varicella encephalitis.

While lumbar puncture remains the gold standard for diagnosing varicella meningoencephalitis, inherent to this invasive procedure are potential risks. These range from minor adverse effects such as post-dural puncture headache and nerve irritation to life-threatening serious complications including infection and hemorrhage (Evans, 1998). Furthermore, overlapping with common respiratory infections and other forms of viral encephalitis are the early clinical manifestations of varicella encephalitis in children, which often lack specific indicators (Carreño et al., 2007; Kennedy, 2023; Zatoulovski et al., 2025). Consequently, in situations where a timely lumbar puncture is not feasible, this clinical ambiguity can readily contribute to diagnostic delays or misdiagnosis.

Machine learning, as a new data mining and statistical analysis technology, brings breakthrough opportunities for disease diagnosis in the medical field. By leveraging powerful algorithmic models, it enables in-depth parsing and pattern mining of complex, multi-dimensional medical data (Aljehani and Al Nawees, 2025). Particularly in the field of central nervous system infections, the application of machine learning has demonstrated significant potential. Studies have integrated clinical symptoms, routine laboratory parameters, and imaging features to construct machine learning models for discriminating between viral and bacterial meningoencephalitis, achieving accuracy rates significantly higher than those of traditional clinical diagnostic methods (Mentis et al., 2021; Victor et al., 2025). Furthermore, by utilizing machine learning algorithms to mine cerebrospinal fluid biomarker data, researchers have provided a novel technical pathway for the early warning and progression assessment of central nervous system infections (Caragheorgheopol et al., 2023). Collectively, these studies lay a solid foundation for the application of machine learning in predicting varicella encephalitis in children.

This study aims to construct a prediction model for varicella encephalitis in children based on machine learning methodologies, thereby providing a reference ffor clinical practice and facilitating its early identification and intervention.

## 2 Patients and methods

### 2.1 Patients and grouping

**Figure 1:**
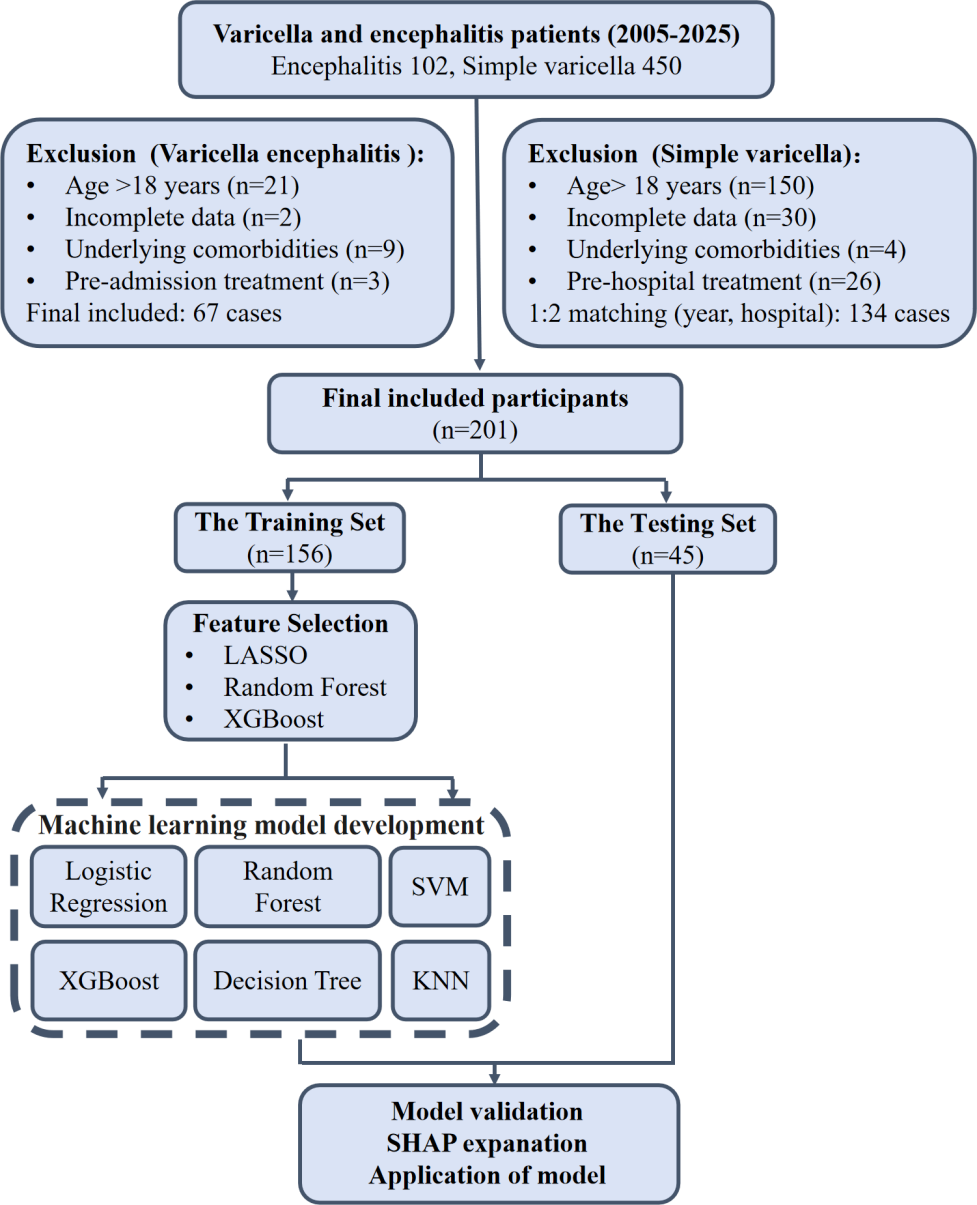
Flowchart of patient selection and machine learning model construction for varicella encephalitis risk prediction.

A retrospective study design was used to select patients hospitalized for chickenpox in Beijing You ‘an Hospital and Beijing Di’tan Hospital from November 2005 to May 2025 (**Figure 1**).

Inclusion criteria were as follows: (1) Varicella encephalitis group: meeting the diagnostic criteria for varicella encephalitis (Tunkel et al., 2008), a confirmed varicella infection accompanied by central nervous system symptoms (such as headache, vomiting, impaired consciousness, seizures, etc.) and supported by findings from neuroimaging (CT/MRI) or cerebrospinal fluid analysis (via lumbar puncture). (2) Simple varicella group: patients with a confirmed varicella infection (based on typical rash presentation and positive varicella-zoster virus antibody testing) but without any central nervous system symptoms or signs, and with other infectious diseases and complications excluded.

Exclusion criteria were: (1) age 18 years or older; (2) receipt of any antiviral treatment prior to hospitalization; (3) presence of severe underlying diseases, such as congenital heart disease, chronic kidney disease, autoimmune diseases, or malignancies; (4) incomplete clinical data.

To control for confounding factors related to year and hospital source, the 1:2 individual matching method was employed. Using each varicella encephalitis patient as a matching core, two simple varicella patients from the same year and the same hospital were randomly selected as controls. Ultimately, 134 simple varicella patients and 67 varicella encephalitis patients were included. Using the year 2015 as a cutoff point, the matched dataset was divided into the training group (November 2005 - December 2015) comprising 156 cases and the testing group (January 2016 - May 2025) comprising 45 cases, for the construction and validation of the machine learning model for varicella encephalitis identification.

This study was approved by the Ethics Committee of Beijing You’an Hospital, Capital Medical University (LL-2025-034-K). All participants have signed written informed consent.

### 2.2 Methods

#### 2.2.1 Data collection

All clinical features and laboratory test indicators in this study were uniformly collected within 48 hours after admission, representing early clinical data obtainable prior to a definitive diagnosis of varicella encephalitis.

Collected for analysis were baseline characteristics from both patient groups, including sex, age, rash duration, thermal course, presence or absence of high fever, presence or absence of vomiting, and presence or absence of skin infection. High fever was defined as a body temperature ≥39°C. The thermal course was defined as the period from the onset of fever (body temperature ≥38°C) to the point when body temperature returned to normal and remained stable for over 24 hours.

Fasting venous blood (5 mL) was collected for the following tests: White blood cell count, neutrophil percentage (N%), and lymphocyte percentage were measured using the electrical impedance method; C-reactive protein was detected by latex-enhanced immunoturbidimetry; and indicators including alanine aminotransferase, aspartate aminotransferase, lactate dehydrogenase, creatine kinase, creatine kinase - myocardial band and hydroxybutyrate dehydrogenase were measured using the enzyme kinetic rate method.

#### 2.2.2 Key variables screening

In the training group, the Least Absolute Shrinkage and Selection Operator (LASSO) regression was employed to screen all clinical and laboratory indicators for key features. The optimal penalty parameter λ (λ 1se) was determined via 10-fold cross-validation using the one standard error (1se) criterion (Kwak, 2025). Features with non-zero coefficients were retained based on this optimal λ1se value, constituting the key variables identified by the LASSO regression screening. Extreme Gradient Boosting (XGBoost) constructs a high-precision ensemble decision tree model by iteratively integrating multiple weak classifiers with lower accuracy, thus demonstrating efficient classification performance. Random Forest builds multiple decision tree instances based on random sampling of the training dataset, through random feature selection and node splitting, it evaluates the importance of each feature and outputs a ranking of feature importance. To enhance variable stability and reliability, thereby further reducing overfitting risk, the intersection of features selected by the three algorithms—LASSO regression, XGBoost, and Random Forest—was taken as the final feature set for modeling.

#### 2.2.3 Univariate analysis and incremental-value validation of the model

For each key feature retained, a univariate receiver-operating-characteristic (ROC) analysis was performed, from which the area under the curve (AUC) together with its 95% confidence interval (CI) was computed; thereby, the diagnostic performance and clinical usefulness of every single variable in discriminating varicella encephalitis were quantified.

To verify the incremental contribution of laboratory indices, a baseline model incorporating only clinical variables was constructed, followed by an extended model that additionally included laboratory indices. By comparing the AUC, Akaike information criterion (AIC), Bayesian information criterion (BIC), Likelihood Ratio Test and Net Reclassification Index (NRI) between the two models, the predictive gain attributable to the laboratory panel and its incremental value over the clinical-only model were systematically assessed and quantified.

#### 2.2.4 Machine learning models development and performance evaluation

Employed for constructing and validating identification models for pediatric varicella encephalitis, based on the selected key variables, were six classical supervised machine learning algorithms: Logistic Regression, Random Forest, Support Vector Machine (SVM), XGBoost, K-Nearest Neighbors (KNN), and Decision Tree. To enhance evaluation robustness and minimize bias from data partitioning, all models were subjected to 10−fold cross−validation and underwent optimism correction via 1000 bootstrap resampling iterations to address overfitting and optimistic bias.

Analyzed via learning curves was the trend of model AUC changes across varying sample sizes. Comprehensively evaluated were the predictive performance of each model using the following metrics, along with their 95% CIs: AUC, accuracy, recall, precision, F1-score, and specificity. Assessed for calibration and reliability of predicted probabilities were calibration curves, the Brier score, and the calibration slope (Alba et al., 2017). Furthermore, quantified using Decision Curve Analysis (DCA) was the clinical net benefit of each model across a range of decision thresholds (Vickers and Elkin, 2006, Rousson and Zumbrunn, 2011, Kerr et al., 2016).

For sensitivity analysis, missing values across variables were handled using mean/mode imputation; the optimal machine learning model was retrained based on the imputed dataset, and the AUC was recalculated. The results demonstrated only minor differences in AUC before and after imputation, with core conclusions remaining consistent, indicating favorable robustness of the findings.

### 2.2.5 Model comparison and webpage deployment tool

To enhance model comparison, SHapley Additive exPlanations (SHAP) values were calculated, allowing for the ranking of feature importance and visualization of directional impacts on model predictions. To facilitate the practical utility of the model in clinical settings, the finalized prediction model was implemented into a web-based application utilizing the Streamlit Python framework. Upon input of the numerical values corresponding to the features in the final model, this application returns an individual’s predicted probability for varicella encephalitis alongside an explanatory SHAP plot.

### 2.3 Statistical analysis

Conducted using SPSS software (version 25.0), the analysis involved the following methods. Categorical data were presented as frequencies (percentages), with between-group comparisons performed using Fisher’s exact test. Continuous data were expressed as the median (P25, P75), and between-group differences were assessed using the Mann-Whitney U test. A two-sided P-value of less than 0.05 was considered statistically significant.

LASSO regression was performed using the “glmnet” package (version 4.0-2) within the R language environment (version 4.2.1). All machine learning model training and evaluation were conducted in Python (version 3.9), utilizing libraries including Scikit-learn, XGBoost, and SHAP.

## 3 Result

### 3.1 Characteristics of the study participants

A total of 201 varicella patients were enrolled from the case systems of You’an Hospital and Dit’an Hospital based on the predefined inclusion and exclusion criteria. Stratified by admission time before or after 2016, the cohort was divided into a training set (n=156, 77.6%) and a testing set (n=45, 22.4%). The prevalence of varicella encephalitis was 33.3% in both the training and testing sets, ensuring comparable risk profiles for model development and validation. Baseline characteristics and laboratory parameters were well-balanced between the two sets, with no significant differences observed in any indicators (all *P* > 0.05, **Table 1**). The absence of significant distribution shifts confirms the rationality and robustness of the dataset for machine learning analysis.

**Table 1:** Baseline characteristics and laboratory examination data of pediatric varicella patients: training vs. testing set comparison.

Variables	Overall (n=201)	The Training Set (n=156)	The Testing Set (n=45)	*P*
Baseline characteristics				
Age(y)	7.00(2.50,11.01)	6.21(2.01,10.43)	9.34(4.05,13.10)	0.077
Gender(%)				0.273
Boy	137(68.2)	110(70.5)	27(60)	
Girl	64(31.8)	46(29.5)	18(40)	
Fever duration(d)	6.00(4.00,7.00)	6.00(4.00,7.00)	5.00(4.50,7.00)	0.491
Hyperpyrexia(%)				0.712
No	11(5.5)	8(5.1)	3(6.7)	
Yes	190(94.5)	148(94.9)	42(93.3)	
Rash duration(d)	8.00(7.00,9.00)	8.00(8.00,9.00)	8.00(7.00,9.00)	0.052
Vomiting(%)				0.185
No	146(72.6)	117(75.0)	29(64.4)	
Yes	55(27.4)	39(25.0)	16(35.6)	
Headache(%)				0.578
No	142(70.6)	112(71.8)	30(66.7)	
Yes	59(29.4)	44(28.2)	15(33.3)	
Laboratory examination				
WBC (×10^9^/L)	6.90(5.58,10.60)	7.17(5.62,11.137)	6(4.62,8.68)	0.066
N (%)	55.90(40.05,68.44)	55.92(38.02,72.77)	55.90(44.70,62.90)	0.786
L (%)	33.90(21.28,46.57)	34.00(19.28,47.16)	32.34(27.50,42.70)	0.647
PLT (×10^9^/L)	197.00(146.00,284.50)	198.50(145.50,274.50)	184.00(146.00,294.50)	0.928
CRP (mg/L)	2.97(0.30,13.00)	2.40(0.01,15.00)	3.4(1.41,10.95)	0.324
ALB (g/L)	23.50(16.45,38.50)	24.40(16.62,39.45)	19.00(14.00,35.35)	0.261
ALT (U/L)	30.80(21.35,44.85)	32.95(21.80,45.35)	30.00(18.00,43.45)	0.102
AST (U/L)	42.50(38.35,45.20)	42.50(39.60,44.97)	42.60(35.95,46.40)	0.530
LDH (mmol/L)	344.00(245.00,394.00)	345.50(243.05,400.60)	337.10(249.10,370.85)	0.454
CK (U/L)	83.60(47.00,143.25)	75.00(45.70,135.60)	122.00(69.50,170.00)	0.059
CK-MB (ng/mL)	20.00(11.00,32.50)	20.50(12.00,33.75)	20.00(6.56,26.90)	0.122
HBDH (U/L)	317.00(255.00,357.00)	314.00(252.00,380.25)	317.00(296.50,32.00)	0.966
CR (μmol/L)	3.65(2.68,4.49)	3.44(2.63,4.44)	4.00(3.24,4.73)	0.111
UA (μmol/L)	39.80(26.65,54.35)	36.6(26.57,52.75)	43.00(27.95,65.00)	0.063
Urea (mmol/L)	233.20(184.00,310.80)	233.55(181.25,311.40)	233.00(186.00,256.50)	0.588
Glu (mmol/L)	5.40(4.86,6.18)	5.44(4.96,6.31)	5.16(4.50,6.04)	0.071

WBC, white blood count; N%, neutrophil percentage; L%, lymphocyte percentage; PLT, platelet count; CRP, C-reactive protein; ALB, albumin; GLB, globulin; ALT, alanine aminotransferase; AST, aspartate aminotransferase; LDH, lactate dehydrogenase; CK, creatine kinase; CK-MB, creatine kinase–myocardial band; HBDH, hydroxybutyrate dehydrogenase; CR, creatinine; UA, uric acid; Urea, urea; Glu, glucose.

Overall, patients with varicella encephalitis were similar in age of onset to children with sample varicella. However, noted in the varicella encephalitis group were a relatively higher proportion of males, significantly longer durations of fever and rash (*P* < 0.001), and significantly higher incidences of vomiting and headache (*P* < 0.001) compared to the sample varicella group (**Supplementary Table 1**). Furthermore, compared to children with sample varicella, those with varicella encephalitis exhibited significantly higher neutrophil percentage, platelet count, C-reactive protein, and glucose levels (*P* < 0.05), alongside a significantly lower lymphocyte percentage (*P* < 0.001) (**Supplementary Table 1**).

### 3.2 Key variables screening

**Figure 2:**
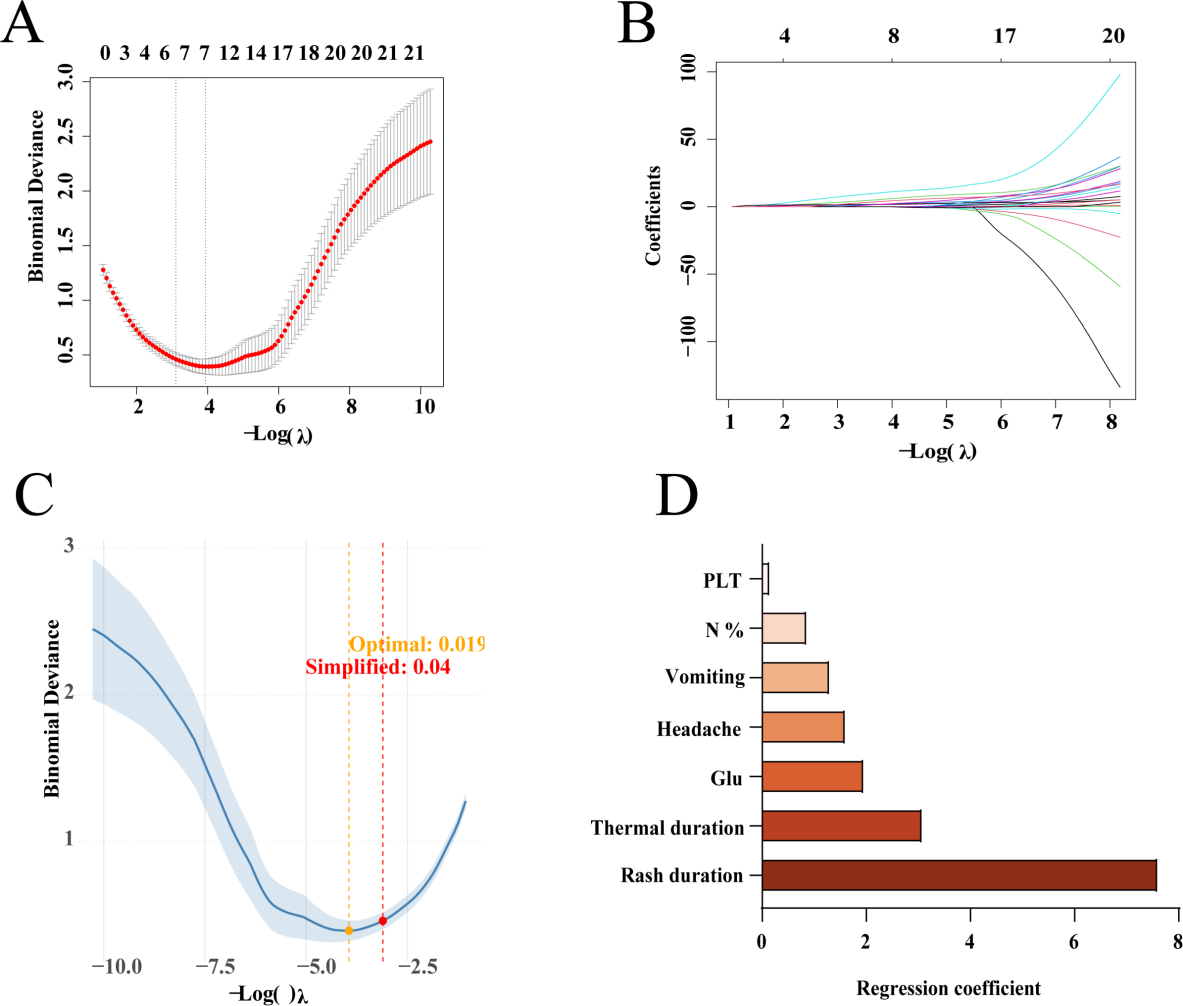
Lasso regression screening for risk factors. **(A)** LASSO binomial deviance curve. **(B)** The screening process of predictive variables (coefficient distribution). **(C)** Calculate the value of λ. **(D)** Non-zero predictive variables selected by Lasso regression screening. **Figure 2A** shows mean square error values and eigenvalues for different λ values. The dashed line on the left is λ-min, meaning λ at the minimum deviation, and the dashed line on the right is λ-se, meaning 1 standard error on the right of the minimum λ. **Figure 2B** shows eigenvalues and residual eigenvalues for different λ values. Each curve represents a feature. The lower and upper abscissa represent different λ values and residual eigenvalues, respectively. The ordinate represents the corresponding coefficient.

Based on the training set, employed for screening key variables was the LASSO regression algorithm, with the penalty parameter iteratively tuned via 10-fold cross-validation (**Figure 2A**). The LASSO coefficient trace plot illustrates the trajectory of coefficients for various clinical and laboratory indicators across a spectrum of λ values (**Figure 2B**). Using the simplified λ value (λ=0.04), the LASSO regression model identified seven clinical and laboratory features with non-zero coefficients (**Figures 2C, D**).

**Figure 3:**
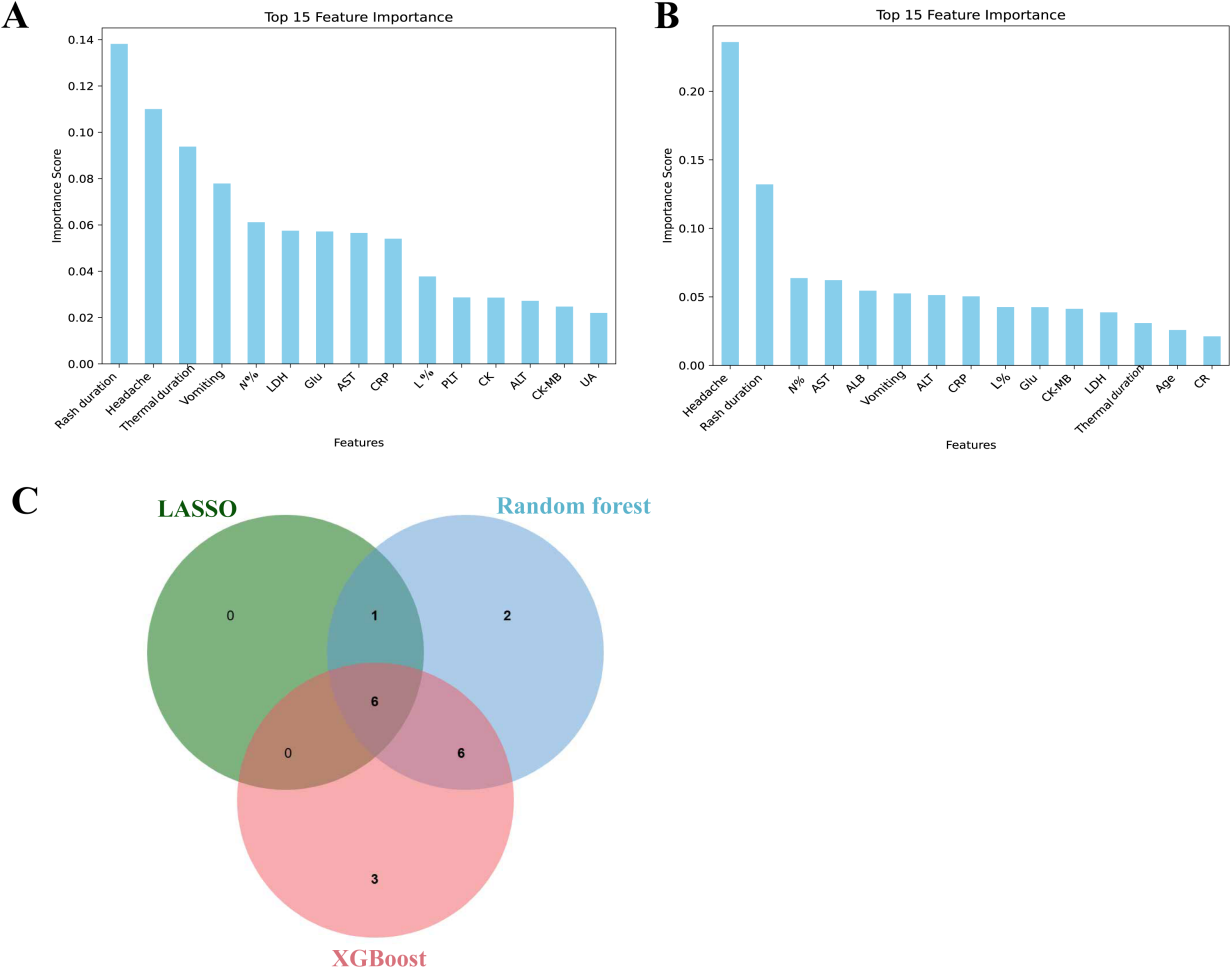
**(A)** The variables selected by Random Forest sorted by importance. **(B)** The variables selected by XGBoost sorted by importance. **(C)** Variable Venn diagram screened by three methods.

The XGBoost model, characterized by strong generalization and good scalability, was employed to determine the top 15 important features (shown in **Figure 3A**). Using an ensemble learning framework, the random forest model showed excellent robustness and high resistance to overfitting, thus generating stable and reliable predictions. The top 15 key features ranked by importance are illustrated in **Figure 3B**.

To improve the stability and reliability of the selected variables, and to establish a refined and highly discriminative feature set for subsequent model development, the intersection of features identified by the three aforementioned algorithms was defined as the final key feature set (**Figure 3C**). The final feature set included: fever duration, rash duration, vomiting, headache, N% and glucose.

### 3.3 Univariate analysis and incremental-value validation of the model

**Figure 4:**
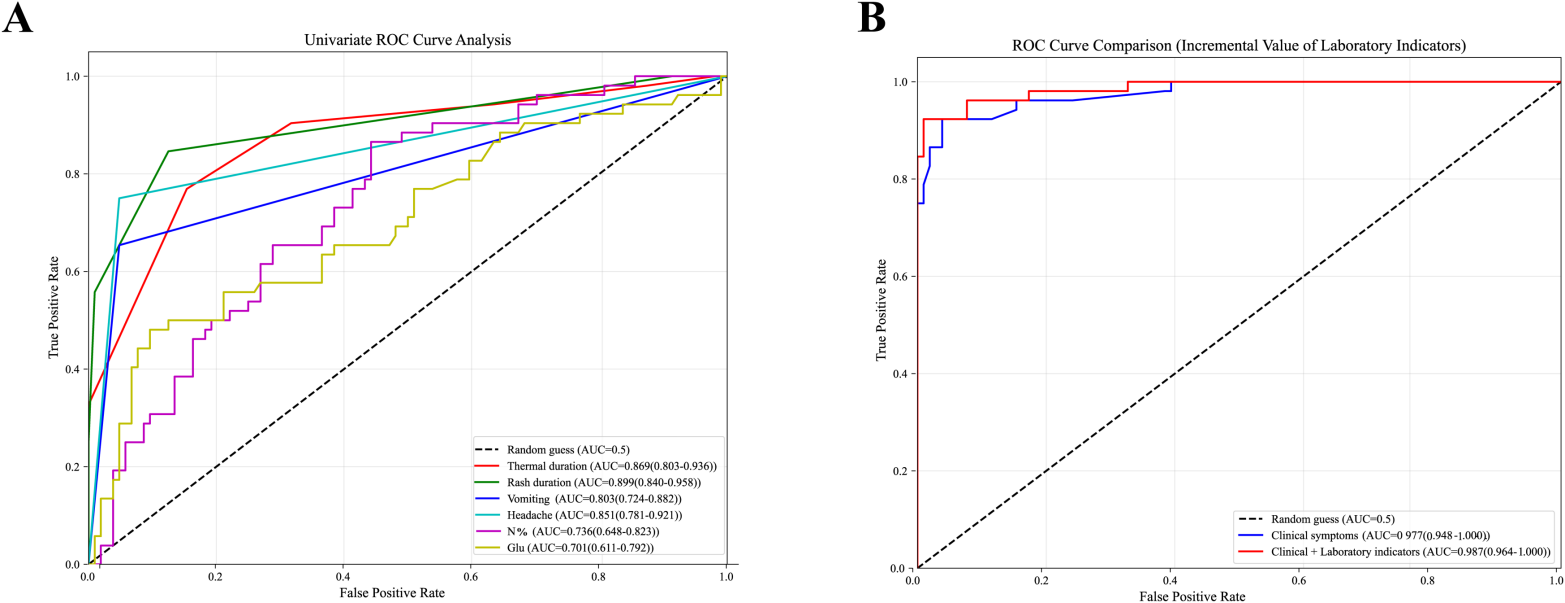
**(A)** Univariate ROC curve analysis of key predictors. **(B)** Incremental value of laboratory indicators in ROC curve comparison. Clinical symptoms including fever duration, rash duration, vomiting, headache. Clinical+ laboratory indicators including fever duration, rash duration, vomiting, headache, Neutrophil percentage and glucose.

Performed on the key feature variables was univariate ROC curve analysis, with the resulting ROC curves illustrated in **Figure 4A**. The analysis revealed that the AUC for individual features ranged from 0.701 to 0.899. Demonstrated was the highest discriminatory ability for the duration of rash (AUC = 0.899, 95% CI: 0.840–0.958), followed by the duration of fever (AUC = 0.869, 95% CI: 0.803–0.936).

Incremental value analysis indicated that the model combining clinical and laboratory indicators provided a superior fit compared to the model containing clinical symptoms alone. This was evidenced by significantly lower values for both AIC (52.19 vs. 62.02) and BIC (73.54 vs. 77.27). The difference between the two models was confirmed to be statistically significant by the likelihood ratio test (χ² = 13.83, *P* = 0.0010). Although the AUC showed only a modest increase from 0.977 to 0.987 (absolute increment 0.010), and the NRI of 0.0288 (95% CI: -0.0652 - 0.1229, *P* = 0.5478) did not reach statistical significance (**Figure 4B**), the introduction of laboratory indicators substantially improved the model’s goodness-of-fit and provided statistically significant incremental information. Therefore, for the subsequent construction of machine learning models, all clinical and laboratory features were included. This approach was adopted to retain potential predictive information and to reflect the comprehensive value of multi-dimensional indicators.

### 3.4 Machine learning models development and performance evaluation

**Figure 5:**
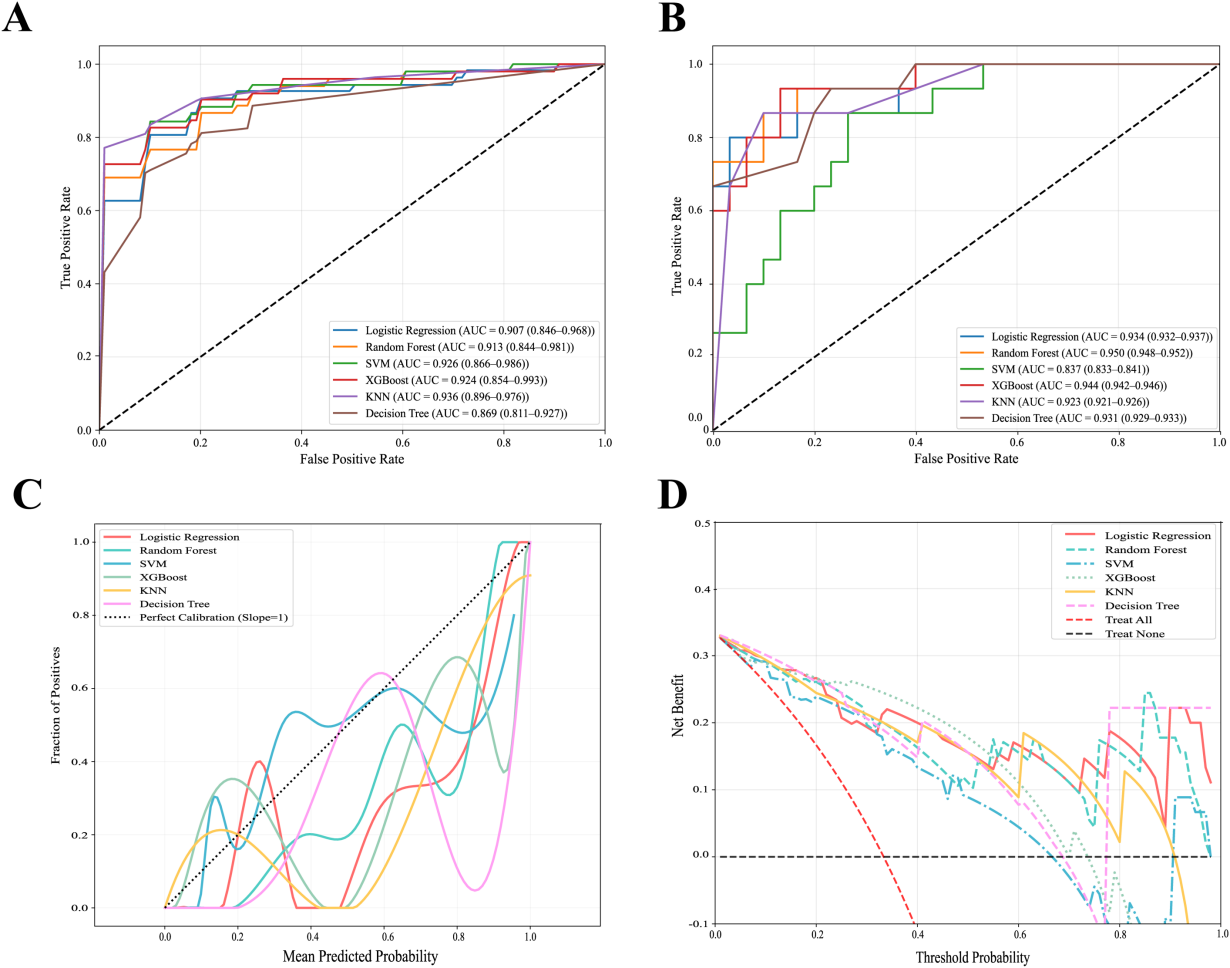
Performance evaluation of multiple machine learning models for predicting pediatric varicella encephalitis. **(A)** Receiver operating characteristic (ROC) curves of various machine learning models in the training set. **(B)**ROC curves of the various machine learning models in the testing set. **(C)** Calibration curve for the prediction model of pediatric varicella encephalitis in testing set. **(D)** Decision curve for the identification model of childhood varicella encephalitis. The area under the curve (AUC) with 95% confidence intervals (95% CI) is presented for each model. SVM, Support Vector Machine; XGBoost, Extreme Gradient Boosting; KNN, K-Nearest Neighbors; SVM, Support Vector Machine.

In the training set, the AUC of each model exhibited an increasing trend with the expansion of the sample size, among which SVM, XGBoost, and Random Forest demonstrated stable performance, consistently maintaining high levels (**Supplementary Figure 1**). The ROC curves from the training set revealed AUCs greater than 0.9 for XGBoost, Random Forest, KNN, SVM, and Logistic Regression, while the Decision Tree performed slightly lower (AUC = 0.869, 95% CI: 0.811–0.927) (**Figure 5A**). After Bootstrap optimism correction, the corrected AUCs for Random Forest and XGBoost remained above 0.9 (**Supplementary Table 2**). Calibration analysis indicated excellent agreement between predicted probabilities and actual observations for both XGBoost (Brier score = 0.004, calibration slope = 1.033) and Random Forest (Brier score = 0.014, calibration slope = 1.090), demonstrating superior calibration (**Supplementary Figure 2**, **Supplementary Table 3**).

In the test set, significantly superior was the generalization performance of Random Forest, achieving an AUC of 0.950 (95% CI: 0.948–0.952). Following were XGBoost (0.944, 95% CI: 0.942–0.946), Logistic Regression (0.934, 95% CI: 0.932–0.937), and Decision Tree (0.931, 95% CI: 0.929–0.933), whereas SVM demonstrated relatively poorer performance (**Figure 5B**). Bootstrap optimism correction results indicated that the highest corrected AUC was attained by Random Forest (0.736, 95% CI: 0.576–0.883) (**Supplementary Table 2**). With a sensitivity of 0.937 (95% CI: 0.933–0.941), equal to that of XGBoost and Decision Tree, Random Forest exhibited outstanding capability in identifying positive events (**Supplementary Table 2**). Well-maintained was the calibration of the Random Forest model in the test set (Brier score = 0.118, calibration slope = 0.985) (**Figure 4C**, **Supplementary Table 3**). DCA revealed that across a wide range of threshold probabilities, significantly higher was the net clinical benefit of Random Forest compared to other models, indicating greater potential for clinical application (**Figure 5D**). Based on this comprehensive multi-dimensional evaluation, selected as the optimal predictive model in this study was Random Forest.

Sensitivity analysis demonstrated robust results, as indicated by an AUC of 0.997 for the Random Forest model after mean/mode imputation for missing values, differing by only 0.047 from the original test set AUC, suggesting that the findings are not substantially influenced by the method of missing data handling.

To determine the optimal risk stratification threshold, the Youden index (J = sensitivity + specificity − 1) was calculated for the Random Forest model across all probability thresholds from 0% to 100%. The maximum Youden index was achieved at a threshold of 61.3%. Accordingly, patients with predicted probabilities ≥ 61.3% were classified as high-risk, and those with probabilities < 61.3% as low-risk, thereby enabling risk stratification for varicella encephalitis patients (**Supplementary Figure 3**).

### 3.5 Model comparison and webpage deployment tool

**Figure 6:**
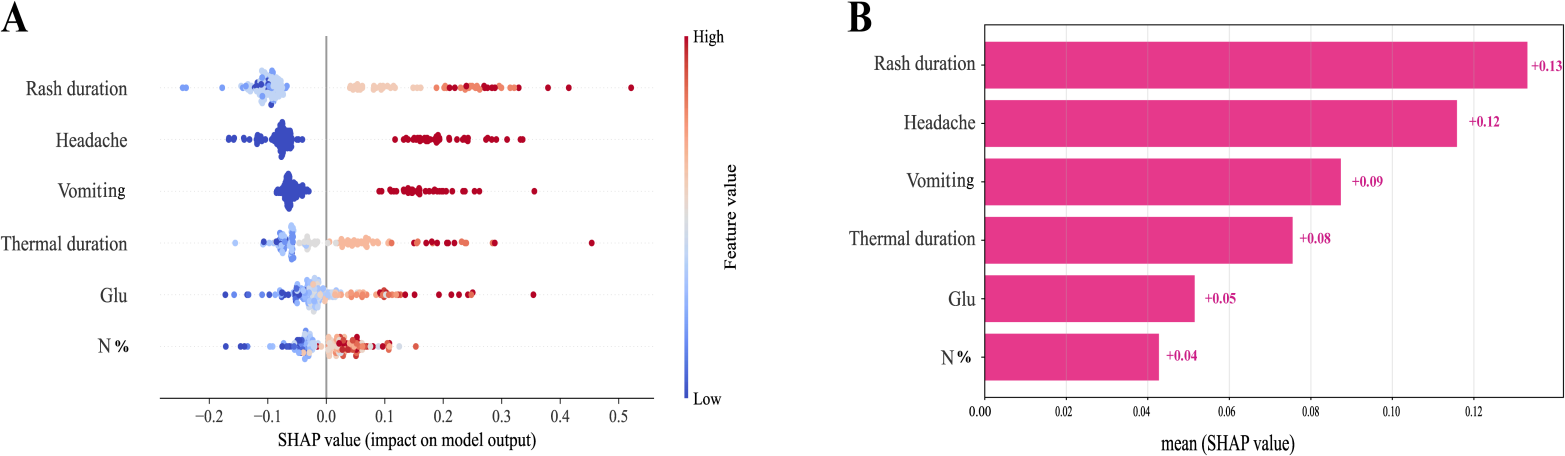
Description of the SHapley Additive exPlanations (SHAP) values and feature importance. **(A)** SHAP value distribution of key predictors. **(B)** Importance score ranking of the model prediction characteristics. **(A)** Every feature’s impact on the model’s output. Every dot in a row symbolizes a patient, and its color denotes the feature value: red denotes a greater value, and blue denotes a lower value. The more dispersed the points of the graph represent the greater the impact of the variables on the model.

To elucidate the contributions of feature variables to the identification of varicella encephalitis, SHAP technology was employed. The SHAP summary plot further revealed the specific impact patterns of individual features (**Figure 6A**): high values for rash duration, headache, and vomiting corresponded to positive SHAP values, significantly increasing the predicted risk of varicella encephalitis, whereas their low values were associated with negative SHAP values, thereby reducing the predicted risk. Elevated levels of fever duration, glucose, and neutrophil percentage also tended to yield positive SHAP values, suggesting that increases in these indicators are associated with a higher predicted risk. The SHAP bar plot indicated that rash duration, headache, and vomiting were the top three most influential features on the model’s predictions (**Figure 6B**).

**Figure 7:**
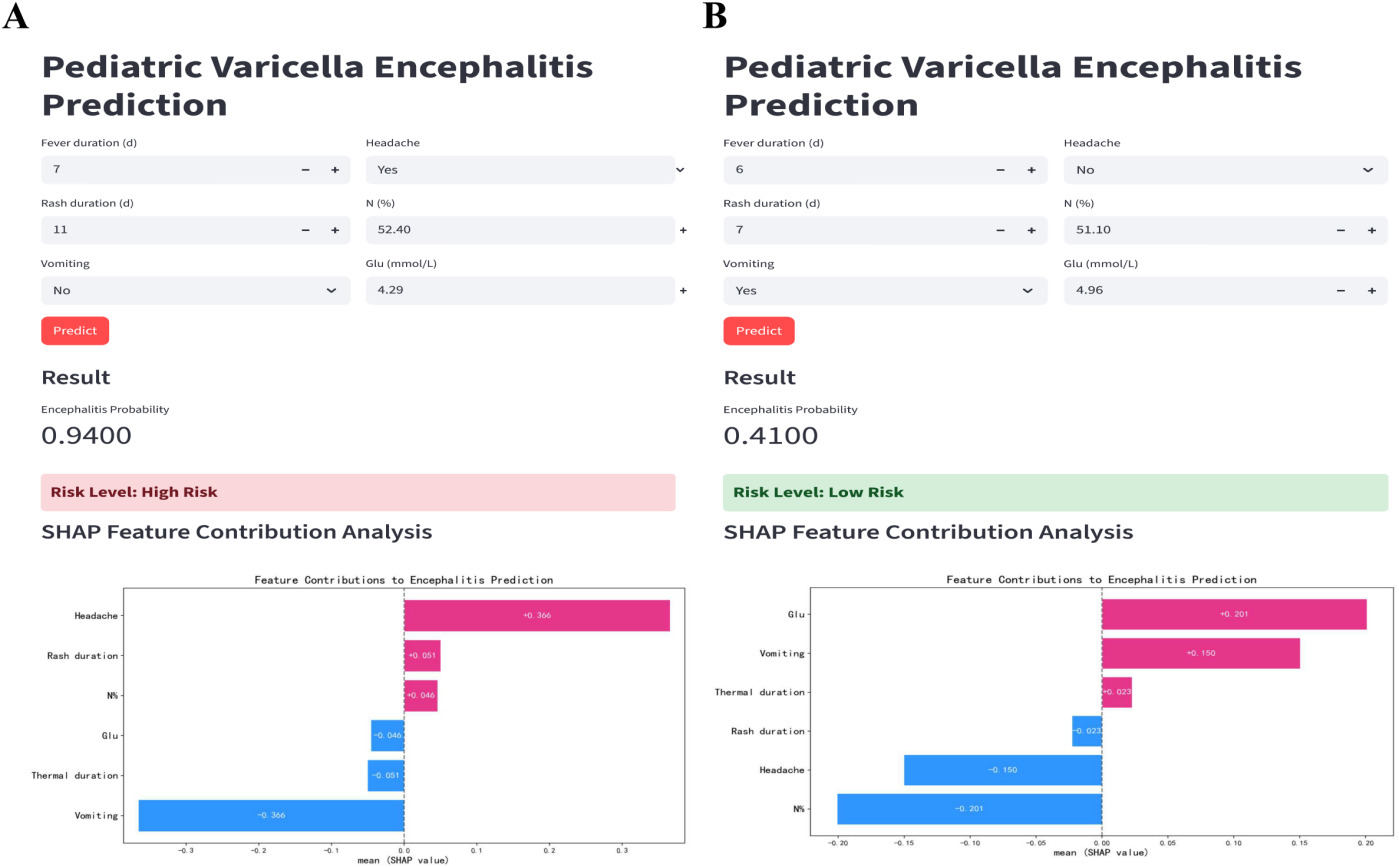
Clinical implementation and interpretability of the machine learning-based prediction system for pediatric varicella encephalitis. **(A)** A representative high-risk case, with a predicted varicella encephalitis probability of 94%. SHAP analysis identified headache, rash duration, and neutrophil percentage as the most influential features driving this high-risk prediction. **(B)** A representative low-risk case, with a predicted varicella encephalitis probability of 41%. SHAP analysis identified glucose levels, vomiting, and fever duration as the most influential features driving this low-risk prediction.

To further enhance accessibility and practical utility, the optimized model was deployed on a dedicated website. This web application is accessible online (https://varicella-encephalitis-prediction-ikbytsekhzxsqgzfw4elg5.streamlit.app/). This user-friendly platform allows clinicians to input patient indicators and assess the risk of varicella encephalitis. To demonstrate the clinical utility of the prediction tool, two representative cases from the testing set were presented. In Case 1, identified as high-risk by the model, an 8-year-old boy with a fever duration of 7 days, rash duration of 11 days, headache, N% of 53.4%, and glucose of 4.29 mmol/L was assigned a predicted probability of 94%. SHAP analysis confirmed headache, rash duration, and N% as the primary risk drivers (**Figure 7A**). In Case 2, classified as low-risk, a 16-year-old boy with a fever duration of 6 days, rash duration of 7 days, history of vomiting, neutrophil percentage of 51.1%, and glucose of 4.96 mmol/L was given a predicted varicella encephalitis probability of 41%. SHAP analysis for this case highlighted glucose, vomiting, and fever duration as the major contributing factors (**Figure 7B**).

## 4 Discussion

This study represents the first integration of clinical variables into a machine learning framework for predicting the risk of varicella encephalitis in children. Demonstrating superior discriminative performance was our Random Forest model, achieving AUC of 0.950 in the testing set. SHAP analysis identified rash duration, headache, vomiting, fever duration, glucose levels, and N% as the primary predictors of varicella encephalitis risk. DCA further confirmed the model’s robustness and highlighted its clinical utility across a broad range of threshold probabilities. Accessible via an online interactive web-based platform, clinicians can input patient indicators to assess varicella encephalitis risk, indicating substantial potential for broader dissemination. These findings collectively emphasize the significant potential held by machine learning models, particularly the Random Forest algorithm, in stratifying risk among patients with varicella encephalitis.

### 4.1 Machine learning implications and comparative analysis

Varicella encephalitis in children represents a severe complication of varicella-zoster virus infection, where early precise identification and risk stratification are critical for improving patient prognosis. Previous studies have predominantly focused on describing epidemiological characteristics and clinical outcomes, with a notable scarcity of systematic predictive modeling (Carreño et al., 2007). Only Ce Wang et al. have reported a four-variable Logistic Regression model incorporating age, vomiting, poor mental status, and rash duration (AUC = 0.955) (Wang et al., 2024). However, this model completely omitted laboratory indicators and did not perform a clinical net benefit analysis, thereby limiting its objectivity and clinical applicability. Addressing these gaps, this study is the first to integrate laboratory parameters with clinical phenotypes and employ six machine learning algorithms to construct a prediction model for pediatric varicella encephalitis. Demonstrated was comparable AUC performance between our Random Forest model and the logistic regression model reported by Ce Wang et al. (0.950 vs. 0.955). Possessing significant clinical net benefit, the Random Forest model underscores its superior utility for decision-making at high-risk thresholds (≥61.3%). Consistent with the literature on its effectiveness in modeling complex, nonlinear clinical data (Jin et al., 2023), the superior performance of the Random Forest model is particularly evident in capturing interaction effects among heterogeneous variables—such as between laboratory indicators and clinical symptoms. Often overlooked by traditional linear models like Logistic Regression, these interactions are crucial (Zhu et al., 2020; Zhang et al., 2022).

Notably robust is the Random Forest model in handling imbalanced medical data (Xu et al., 2020)—a common challenge in predicting pediatric varicella encephalitis, given its low incidence among children with varicella infection, which readily leads to imbalanced sample sets. Observed with ensemble methods (RF/XGBoost), compared to simpler linear models such as Logistic Regression, was a distinct advantage, lending support to the hypothesis that the risk of developing varicella encephalitis in children is driven by multifactorial, non-additive interactions. Nevertheless, the competitive AUC of the logistic regression model (0.934) suggests that linear relationships may still dominate certain risk pathways, indicating a need for future research into hybrid modeling strategies to further enhance predictive performance and clinical applicability.

Not only did the SHAP analysis identify the core predictors of pediatric varicella encephalitis, but it also quantitatively delineated the direction and magnitude of each variable’s contribution to the risk score, thereby providing intuitive and interpretable insights for clinical decision-making (Lundberg et al., 2020). Successfully applied in related studies on autoimmune encephalitis and Parkinson’s disease (Guo et al., 2025; Fırat, 2026), similar interpretable machine learning strategies further support the promising application and generalizability of our approach in the context of infectious neurological disorders.

### 4.2 Interpretation of key risk factors

Identified as the strongest predictor of pediatric varicella encephalitis was the duration of rash, wherein a longer rash duration correlated with a higher risk of progression to encephalitis—a finding consistent with previous research (Wang et al., 2024). Both the duration of fever and the duration of rash serve as key indicators reflecting the length of viral replication *in vivo*. Positively associated with a longer replication time is a higher viral load in the blood, thereby increasing the probability of the virus crossing the blood-brain barrier. Indicative of potential breach of the blood-brain barrier by the virus, vomiting and headache represent early clinical signals of central nervous system involvement, with vomiting also recognized in the literature as a significant warning indicator (Wang et al., 2024).

Also emerging as an important predictor was an elevated neutrophil count, which suggests a robust inflammatory response and activation of innate immunity. Concurrently, the stress response triggered by severe infection can manifest as elevated blood glucose level. Although not meeting the diagnostic threshold for stress-induced hyperglycemia in adults, glucose levels in the encephalitis group were significantly higher than those in the sample varicella group. Supported by substantial evidence is the association between stress-induced hyperglycemia and adverse outcomes in acute central nervous system injuries, with mechanisms involving metabolic dysregulation, inflammatory activation, and direct neurotoxicity from oxidative stress (Capes et al., 2001; Weiss et al., 2010; Costea et al., 2020).

Following infection of human cerebrovascular fibroblasts, fibroblasts, and other cells by the varicella-zoster virus is a significant increase in IL-6 levels, fostering a pro-inflammatory, tumor microenvironment-like pathological state that exacerbates neural tissue damage (Jones et al., 2017). Playing a crucial role in infection- and inflammation-related pathology is immune surveillance mediated by natural killer cells, whose regulatory patterns share similarities with mechanisms observed within the tumor microenvironment (Saadh et al., 2024b). Involved in the regulation of inflammation, autophagy, and therapeutic response through multiple signaling pathways are long non-coding RNAs, which exhibit complex and debated roles in drug resistance and disease progression (Saadh et al., 2023, 2024a). From a clinical translation perspective, the principles pursued by nano-targeted therapies—such as precise delivery, barrier penetration, and optimization for clinical application—also offer a referential framework for the early intervention of infection-related brain injury (Chehelgerdi et al., 2023).

### 4.3 Clinical integration and workflow implications

In the clinical management pathway for pediatric varicella encephalitis, currently lacking is a quantifiable predictive tool that can be readily deployed in outpatient or emergency settings. Our constructed machine learning model holds the potential to translate theoretical risk stratification into a clinically applicable workflow: by inputting routine clinical data, an individualized risk score can be generated via an interactive web interface, supporting further investigations such as cerebrospinal fluid analysis for high-risk patients (risk probability ≥ 61.3%), thereby enabling early and proactive targeted intervention and making management strategies more personalized and precise. Integrated into this process is SHAP-based interpretability analysis, which visually presents the dominant risk drivers for each individual patient. This workflow can assist clinicians in rapidly performing risk stratification even during stages when symptoms are not yet typical, and promptly initiate confirmatory procedures such as cerebrospinal fluid studies or neuroimaging, ultimately achieving the clinical goals of shortening the diagnostic-therapeutic window, reducing the incidence of severe disease, and lowering disability rates.

### 4.4 Limitations and future directions

First, as a retrospective study with a limited sample size and long study period, we performed 1:2 matching by admission year and hospital to reduce temporal and institutional biases. However, selection bias from incomplete medical records and variations in clinical practice may still exist, limiting the model’s external generalizability. Second, the lack of dynamic time-series data, neuroimaging, and cerebrospinal fluid results may lead to incomplete risk prediction. Third, the limited sample and feature dimensions carry a risk of overfitting. Therefore, this model is exploratory and requires further external validation for clinical application.

Urgently required are large-scale, multi-center prospective studies to independently validate the model’s stability and transferability using external cohorts. Future efforts should further integrate dynamic time-series data, neuroimaging features, and potential molecular biomarkers to mitigate biases and enhance model robustness, ultimately facilitating its translation into clinical practice.

## 5 Conclusion

This study identified six significant clinical variables for pediatric varicella encephalitis and developed a high-accuracy predictive model based on the random forest algorithm. Potentially serving as an early screening tool in clinical practice, this model may assist pediatricians in rapidly identifying children at high risk of varicella encephalitis, enabling timely intervention and reducing the incidence of complications.

## Data Availability

The original contributions presented in the study are included in the article/**Supplementary Material**. Further inquiries can be directed to the corresponding author.

## Supplementary material

Supplementary Figure 1 Learning curve of various machine learning models in the training set.

Supplementary Figure 2 Calibration curve for the prediction model of pediatric varicella encephalitis in the training set.

Supplementary Figure 3 Risk group distribution based on the Youden’s index.
