# Supplementary figures and images for: An explainable machine learning model predicts pediatric varicella encephalitis
Source: Front Cell Infect Microbiol. 2026 Apr 15;16:1759109. doi: 10.3389/fcimb.2026.1759109 (PMC13125112; doi:10.3389/fcimb.2026.1759109)

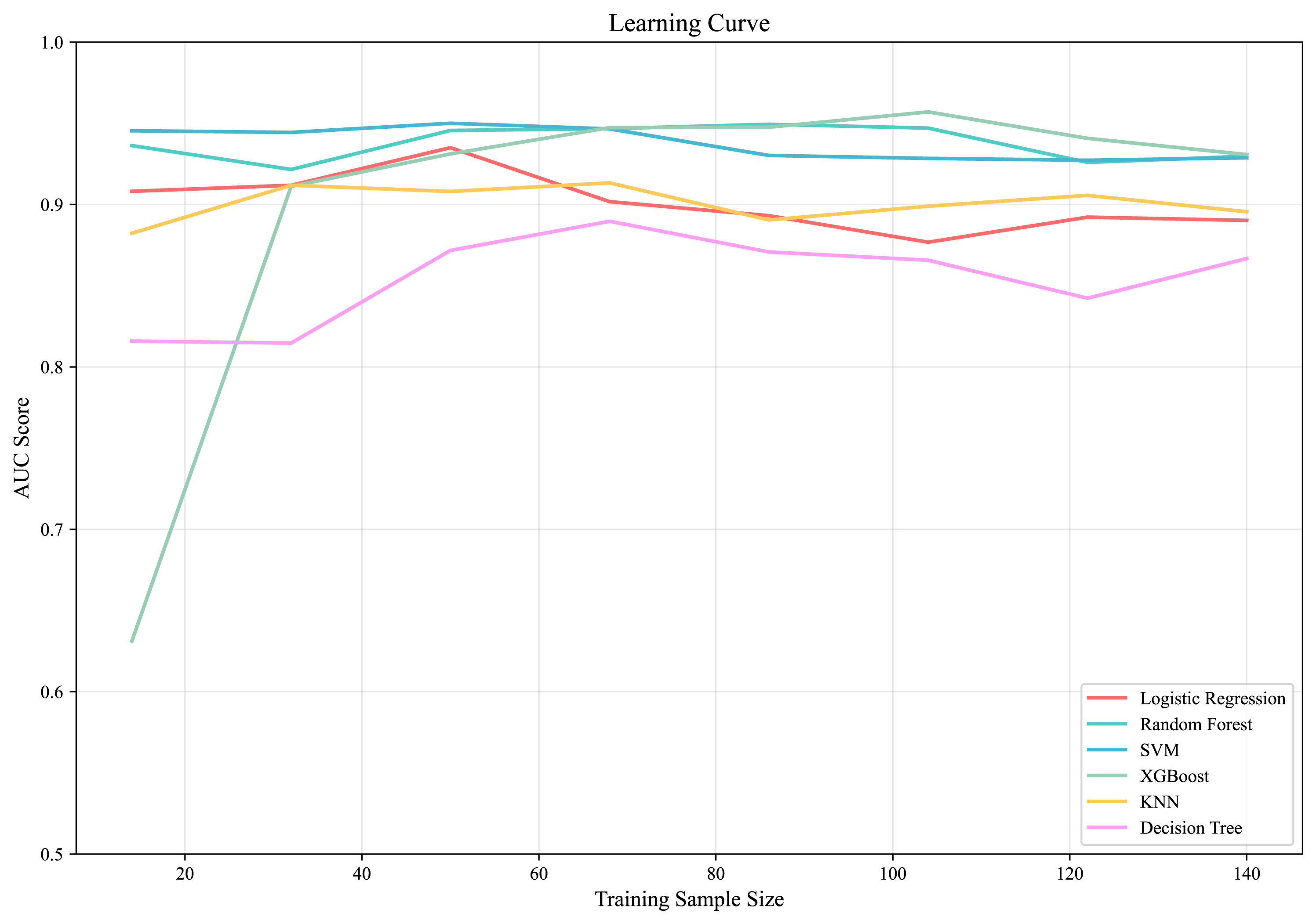

Supplement: Supplementary Figure 1 — Learning curve of various machine learning models in the training set. [file Image1.tif]

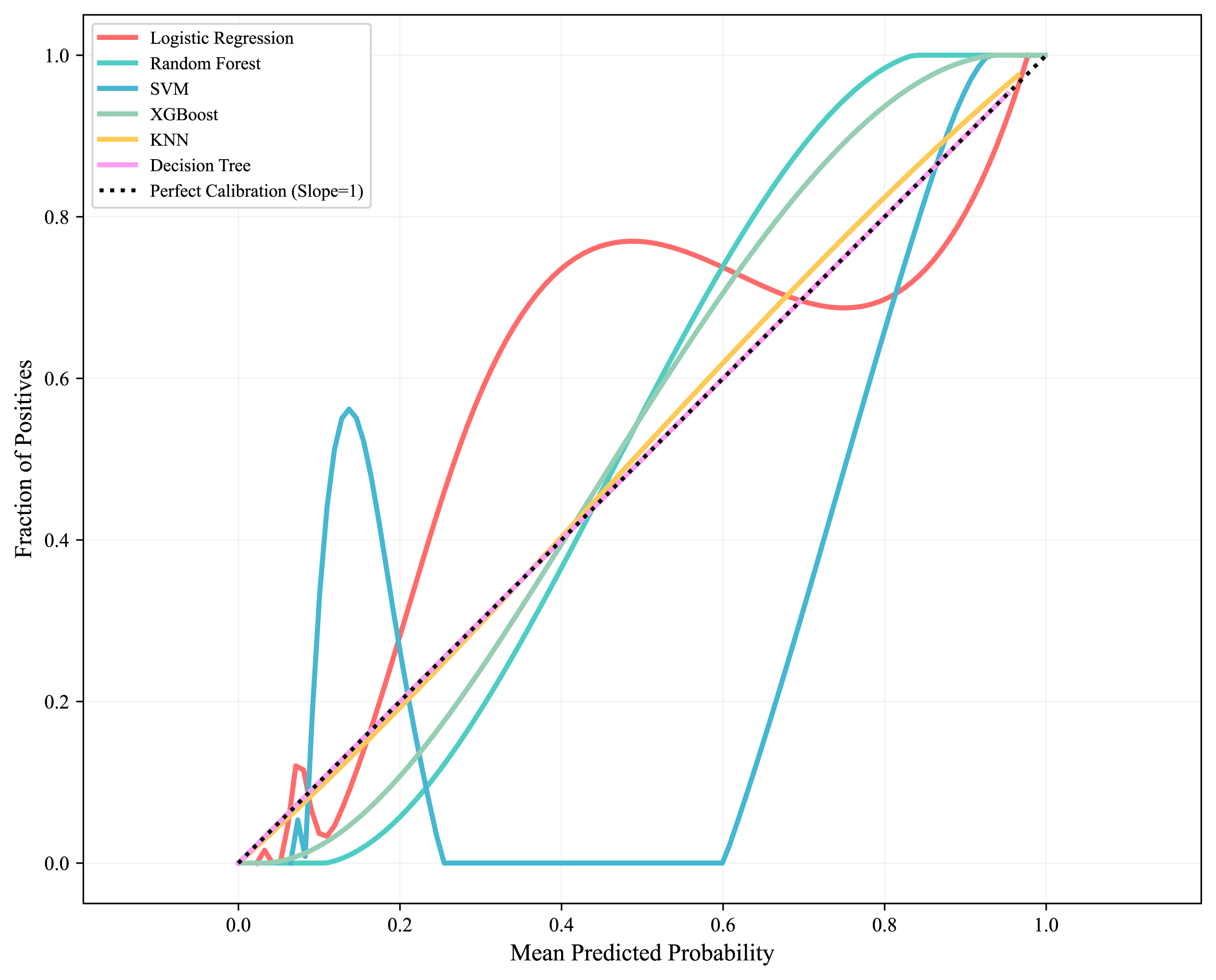

Supplement: Supplementary Figure 2 — Calibration curve for the prediction model of pediatric varicella encephalitis in the training set. [file Image2.tif]

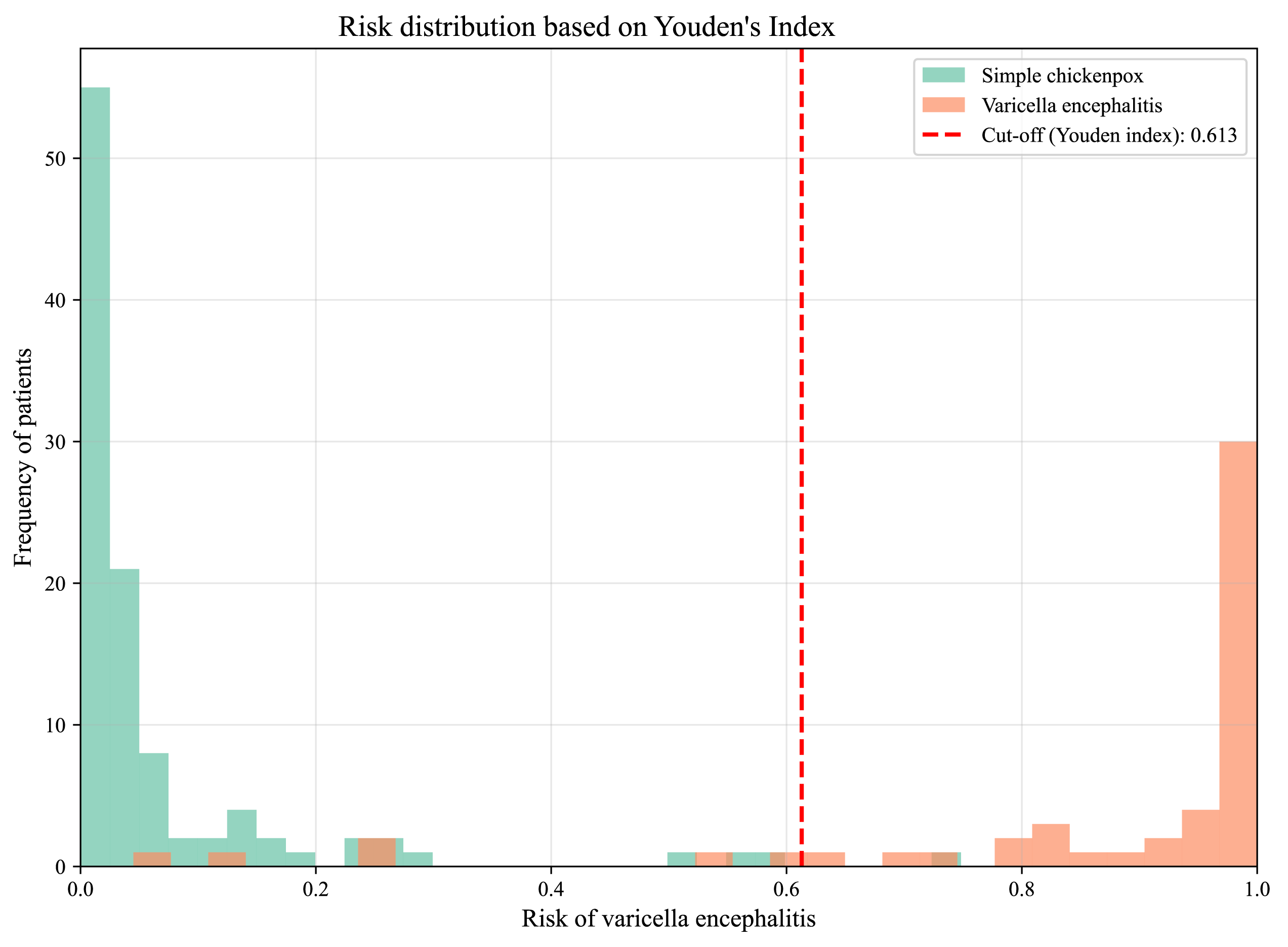

Supplement: Supplementary Figure 3 — Risk group distribution based on the Youden’s index. [file Image3.tif]
