# Supplementary material for: An explainable machine learning model predicts pediatric varicella encephalitis
Source: Front Cell Infect Microbiol. 2026 Apr 15;16:1759109. doi: 10.3389/fcimb.2026.1759109 (PMC13125112; doi:10.3389/fcimb.2026.1759109)
Supplement: Supplementary file 4 [file Table1.docx]

**Table S1 Comparison of baseline characteristics and laboratory examination data in the simple varicella group and the varicella encephalitis group**

| **Variables** | **Overall (n=201)** | **Simple varicella group(*n=134*)** | **Varicella encephalitis group (*n=67*)** | ***P*** |
| --- | --- | --- | --- | --- |
| **Baseline characteristics** |  |  |  |  |
| Age(y) | 7.56(5.22,10.12) | 7.04(4.12,12.02) | 8.00(2.56,13.23) | 0.610 |
| Gender(%) |  |  |  | 0.024 |
| Boy | 137(68.2) | 84(62.7) | 53(79.1) |  |
| Girl | 63(31.8) | 50(37.3) | 14(20.9) |  |
| Fever duration（d） | 7(7,8) | 6(4,7) | 5(4,6) | ＜0.001 |
| Hyperpyrexia（%） |  |  |  | 0.060 |
| No | 27(13.4) | 15(11.2) | 2(3.0) |  |
| Yes | 184(86.6) | 119(88.8) | 65(97.0) |  |
| Rash duration(d) | 10(9,11) | 8(7,9) | 8(7,8) | ＜0.001 |
| Vomiting(%) |  |  |  | ＜0.001 |
| No | 146(72.6) | 124(92.5) | 22(32.8) |  |
| Yes | 55(27.4) | 10(7.5) | 45(67.2) |  |
| Headache(%) |  |  |  | ＜0.001 |
| No | 142(70.6) | 127(94.8) | 15(22.4) |  |
| Yes | 59(29.3) | 7(5.2) | 52(77.6) |  |
| **Laboratory examination** |  |  |  |  |
| WBC  (×10^9^/L) | 6.58(5.47,10.02) | 6.38(4.80,9.52) | 7.29(5.82,11.24) | 0.020 |
| N(%) | 55.90(40.05,68.44) | 50.30(36.83,64.52) | 62.90(50.54,76.31) | ＜0.001 |
| L(%) | 33.90(21.28,46.58) | 35.80(25.2251.10) | 27.00(13.50,40.48) | ＜0.001 |
| PLT  (×10^9^/L) | 197.00(146.00,284.50) | 184.50(137.57.00,239.83) | 273.00(176.00,353.30) | ＜0.001 |
| CRP  (mg/L) | 2.98(0.30,13.00) | 2.00(0.01,13.47) | 3.00(0.09,13.00) | 0.035 |
| ALB  (g/L) | 23.50(16.45,38.50) | 23.20(16.67,39.90) | 23.60(14.00,33.10) | 0.731 |
| ALT  (U/L) | 30.80(21.35,44.85) | 21.20(15.70,40.20) | 33.70(25.48,46.30) | ＜0.001 |
| AST  (U/L) | 42.50(38.35,45.20) | 41.60(36.80,44.10) | 43.00(39.70,45.60) | 0.005 |
| LDH  (mmol/L) | 344.00(245.00,394.00) | 355.00(263.50,405.62) | 263.50(229.10,358.60) | 0.001 |
| CK  (U/L) | 83.60(47.00,154.10) | 87.00(58.62,154.15) | 54.10(41.00,136.20) | 0.188 |
| CK-MB  (ng/mL) | 20.00(11.00,32.50) | 24.00(12.00,33.00) | 18.00(8.00,31.40) | 0.075 |
| HBDH  (U/L) | 317.00(255.00,357.00) | 326.00(283.00,375.75) | 275.00(243.00,327.00) | 0.001 |
| CR  (μmol/L) | 3.65(2.675,4.49) | 3.41(2.625,4.405) | 3.97(2.72,4.85) | 0.078 |
| UA  (μmol/L) | 39.80(26.65,54.35) | 39.75(25.78,57.25) | 39.80(28.40,52.00) | 0.977 |
| Urea  (mmol/L) | 233.20(184.00,310.80) | 233.00(185.00,287.45) | 255.10(178.00,366.00) | 0.105 |
| Glu (mmol/L) | 5.30(4.79,6.03) | 5.16(4.50,5.71) | 5.83(5.13,7.21) | ＜0.001 |

Note: WBC: white blood count, N%: Neutrophil percentage, L%: lymphocyte percentage, PLT: platelet count, CRP: C-reactive protein, ALB: albumin, GLB: globulin, ALT: alanine aminotransferase, AST: aspartate aminotransferase, LDH: lactate dehydrogenase, CK: creatine kinase, CK-MB: creatine kinase- - myocardial band, HBDH: hydroxybutyrate dehydrogenase, CR: creatinine, UA: uric acid, Urea: urea, Glu: glucose.

**Table S2 Performances of the machine learning models for Recognizing pediatric varicella encephalitis.**

|  | **AUC (95%CI)** | **Accuracy (95%CI)** | **Sensitivity (95%CI)** | **Precision (95%CI)** | **F1 (95%CI)** | **Specificity (95%CI)** | **Corrected Accuracy (95%CI)** | **Corrected AUC (95%CI)** |
| --- | --- | --- | --- | --- | --- | --- | --- | --- |
| **The training set** |  |  |  |  |  |  |  |  |
| Logistic Regression | 0.907 (0.846–0.968) | 0.877 (0.808–0.947) | 0.767 (0.603–0.930) | 0.851 (0.723–0.980) | 0.794 (0.663–0.926) | 0.934 (0.879–0.988) | 0.948 (0.917–0.981) | 0.658 (0.464–0.810) |
| Random Forest | 0.913 (0.844–0.981) | 0.839 (0.771–0.907) | 0.747 (0.593–0.900) | 0.757 (0.652–0.861) | 0.745 (0.622–0.867) | 0.885 (0.840–0.929) | 0.949 (0.929–0.968) | 0.913 (0.758–0.999) |
| SVM | 0.926 (0.866–0.986) | 0.897 (0.835–0.959) | 0.807 (0.671–0.942) | 0.875 (0.773–0.977) | 0.832 (0.720–0.945) | 0.942 (0.893–0.991) | 0.945 (0.917–0.981) | -0.121 (-0.365–0.248) |
| XGBoost | 0.924 (0.854–0.993) | 0.871 (0.817–0.925) | 0.767 (0.634–0.900) | 0.833 (0.735–0.931) | 0.789 (0.688–0.889) | 0.924 (0.879–0.968) | 0.980 (0.962–0.994) | 0.905 (0.719–0.999) |
| KNN | 0.936 (0.896–0.976) | 0.903 (0.854–0.953) | 0.787 (0.660–0.913) | 0.922 (0.848–0.996) | 0.837 (0.741–0.933) | 0.960 (0.923–0.997) | 0.975 (0.949–0.995) | 0.836 (0.666–1.000) |
| Decision Tree | 0.869 (0.811–0.927) | 0.819 (0.736–0.902) | 0.647 (0.495–0.799) | 0.788 (0.631–0.945) | 0.697 (0.556–0.839) | 0.904 (0.829–0.978) | 0.956 (0.917–0.987) | 0.845 (0.663–0.984) |
| **The testing set** |  |  |  |  |  |  |  |  |
| Logistic Regression | 0.934 (0.932–0.937) | 0.822 (0.818–0.825) | 0.805 (0.799–0.812) | 0.705 (0.697–0.712) | 0.745 (0.740–0.751) | 0.830 (0.826–0.835) | 0.711 (0.578–0.844) | 0.435 (0.266–0.651) |
| Random Forest | 0.950 (0.948–0.952) | 0.776 (0.772–0.780) | 0.937 (0.933–0.941) | 0.607 (0.600–0.613) | 0.731 (0.726–0.736) | 0.696 (0.691–0.702) | 0.711 (0.578–0.844) | 0.736 (0.576–0.883) |
| SVM | 0.837 (0.833–0.841) | 0.753 (0.749–0.757) | 0.532 (0.524–0.540) | 0.661 (0.652–0.669) | 0.581 (0.574–0.588) | 0.863 (0.859–0.867) | 0.711 (0.578–0.844) | 0.688 (0.529–0.841) |
| XGBoost | 0.944 (0.942–0.946) | 0.844 (0.840–0.847) | 0.937 (0.933–0.941) | 0.698 (0.691–0.705) | 0.795 (0.790–0.800) | 0.797 (0.793–0.802) | 0.689 (0.556–0.822) | 0.615 (0.424–0.797) |
| KNN | 0.923 (0.921–0.926) | 0.821 (0.818–0.825) | 0.870 (0.865–0.876) | 0.683 (0.676–0.690) | 0.760 (0.755–0.765) | 0.797 (0.793–0.802) | 0.711 (0.577–0.844) | 0.394 (0.297–0.510) |
| Decision Tree | 0.931 (0.929–0.933) | 0.822 (0.819–0.826) | 0.937 (0.933–0.941) | 0.666 (0.659–0.672) | 0.773 (0.769–0.778) | 0.765 (0.760–0.770) | 0.733 (0.600–0.867) | 0.620 (0.487–0.750) |

Note: SVM: Support Vector Machine, XGBoost: Extreme Gradient Boosting, KNN: K-Nearest Neighbors, SVM:Support Vector Machine, AUC: area under the curve. CI: confidence interval. Corrected Accuracy and Corrected AUC were estimated using 1000 bootstrap resampling with optimism correction.

**Table S3 Calibration curve indicators for training and testing groups**

|  | **The training set** | | |  | **The testing set** | | |
| --- | --- | --- | --- | --- | --- | --- | --- |
|  | **Brier score** | **Calibration slope** | **Calibration intercept** |  | **Brier score** | **Calibration slope** | **Calibration intercept** |
| XGBoost | 0.004 | 1.033 | -0.011 |  | 0.129 | 0.792 | -0.023 |
| Random Forest | 0.014 | 1.090 | -0.031 |  | 0.118 | 0.985 | -0.12 |
| Decision Tree | 0.048 | 1.000 | 0.000 |  | 0.124 | 0.868 | -0.107 |
| KNN | 0.056 | 1.016 | -0.003 |  | 0.120 | 0.834 | -0.079 |
| SVM | 0.057 | 1.067 | -0.027 |  | 0.165 | 0.756 | 0.053 |
| Logistic Regression | 0.079 | 0.986 | 0.005 |  | 0.111 | 0.954 | -0.085 |

Note: SVM: Support Vector Machine, XGBoost: Extreme Gradient Boosting, KNN: K-Nearest Neighbors, SVM:Support Vector Machine.
